# Comprehensive description of the current breast cancer microenvironment advancements via single-cell analysis

**DOI:** 10.1186/s13046-021-01949-z

**Published:** 2021-04-27

**Authors:** Xueqi Yan, Yinghong Xie, Fan Yang, Yijia Hua, Tianyu Zeng, Chunxiao Sun, Mengzhu Yang, Xiang Huang, Hao Wu, Ziyi Fu, Wei Li, Shiping Jiao, Yongmei Yin

**Affiliations:** 1 Department of Oncology, the First Affiliated Hospital of Nanjing Medical University, Nanjing, 210029 China; 2 Department of Hepatobiliary Surgery, the Affiliated Drum Tower Hospital of Nanjing University Medical School, Nanjing, 210029 Jiangsu Province China; 3 Drum Tower Institute of clinical medicine, Nanjing University, Nanjing, 210029 Jiangsu Province China; 4 Jiangsu Key Lab of Cancer Biomarkers, Prevention and Treatment, Collaborative Innovation Center for Personalized Cancer Medicine, Nanjing Medical University, Nanjing, 211166 China

**Keywords:** Breast cancer, Tumor microenvironment, Single-cell RNA sequencing, Single-cell DNA sequencing

## Abstract

Breast cancer is a heterogeneous disease with a complex microenvironment consisting of tumor cells, immune cells, fibroblasts and vascular cells. These cancer-associated cells shape the tumor microenvironment (TME) and influence the progression of breast cancer and the therapeutic responses in patients. The exact composition of the intra-tumoral cells is mixed as the highly heterogeneous and dynamic nature of the TME. Recent advances in single-cell technologies such as single-cell DNA sequencing (scDNA-seq), single-cell RNA sequencing (scRNA-seq) and mass cytometry have provided new insights into the phenotypic and functional diversity of tumor-infiltrating cells in breast cancer. In this review, we have outlined the recent progress in single-cell characterization of breast tumor ecosystems, and summarized the phenotypic diversity of intra-tumoral cells and their potential prognostic relevance.

## Background

**Fig. 1:**
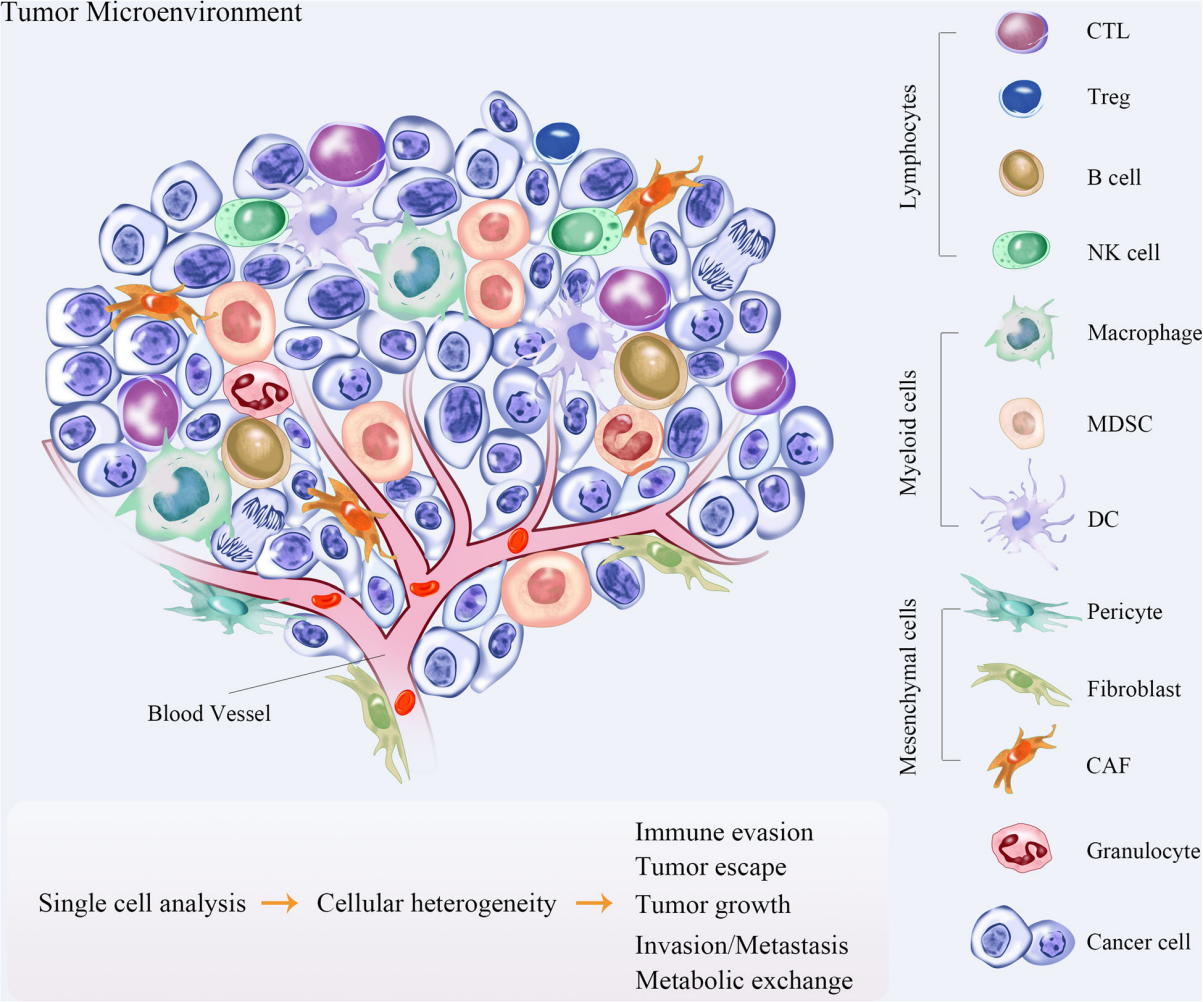
Cellular infiltrates within the TME. Solid tumors harbor CAFs, immune cells and pericytes in the stroma that form a complex regulatory network, which fosters tumor growth by evading immune surveillance

Breast cancer is the major cause of cancer-related deaths among women worldwide [1]. The development of targeted therapies for breast cancer is impeded by the lack of understanding of its tumor microenvironment (TME), a complex ecosystem comprising of cancer cells, immune cells, and stromal populations including fibroblasts and vascular cells (Fig. 1) [2]. Furthermore, both tumor and tumor-associated cells are phenotypically and functionally heterogeneous due to genetic and non-genetic factors. The molecular targets of current therapies, such as the estrogen receptor (ER), human epidermal growth factor receptor 2 (HER2), the phosphatidylinositol 3-kinase (PI3K), the AKT serine/threonine kinases (AKTs), the mammalian target of rapamycin (mTOR), the poly (ADP-ribose) polymerase (PARP), cyclin dependent kinase 4 (CDK4), CDK6 and methyltransferases, also exhibit considerable intra- and inter-patient variations in their expression levels [3]. The heterogeneity of cancer cells enhances their proliferation, survival and invasion, and is the determining factor underlying the differential treatment efficacies [4].

The interactions between the cancer cells and immune cells in the TME promotes immune evasion and tumor growth [5]. For instance, tumor cells, tumor-associated macrophages (TAMs) and stromal cells induce T cell exhaustion by activating co-inhibitory receptors such as PD-1, CTLA-4 and TIM-3. In addition, the regulatory T cells (Tregs) secrete immunosuppressive cytokines that inhibit the tumor-specific cytotoxic T lymphocytes (CTLs) [6]. TAMs can promote tumor growth, invasion and angiogenesis by upregulating the PD-1 ligand (PD-L1) on tumor cells [6, 7]. Therefore, reversal of the immunosuppressive TME is a promising anti-tumor therapeutic strategy, and several immune checkpoint inhibitors (ICIs) that target exhausted and regulatory T cells have been developed in recent years [8, 9]. However, ongoing clinical trials suggest that breast cancer patients respond poorly to immune checkpoint blockade (ICB) compared to melanoma or lung cancer patients, likely due to the lower immunogenicity of breast cancer cells [10]. Interestingly, higher overall response rates have been reported in patients with PD-L1^+^ breast tumors [11]. Given the cellular heterogeneity of breast tumors, patient classification and treatment should ideally consider the entire tumor ecosystem. However, breast tumors are presently stratified on the basis of ER, progesterone receptor (PR), HER2 and the proliferation marker Ki-67 [12] into the luminal A, luminal B, HER2-positive and triple-negative subtypes [13]. In addition, alternative classification schemes based on gene expression and genomic alterations have also been proposed [14], and pathological tumor grading that assesses the morphological deviation of tumor tissues from the normal tissues can predict patient prognosis [15]. Although these stratifications have improved therapeutic outcomes, patient responses still vary within each subtype, thus calling for better characterization of the breast tumor ecosystems.

**Fig. 2:**
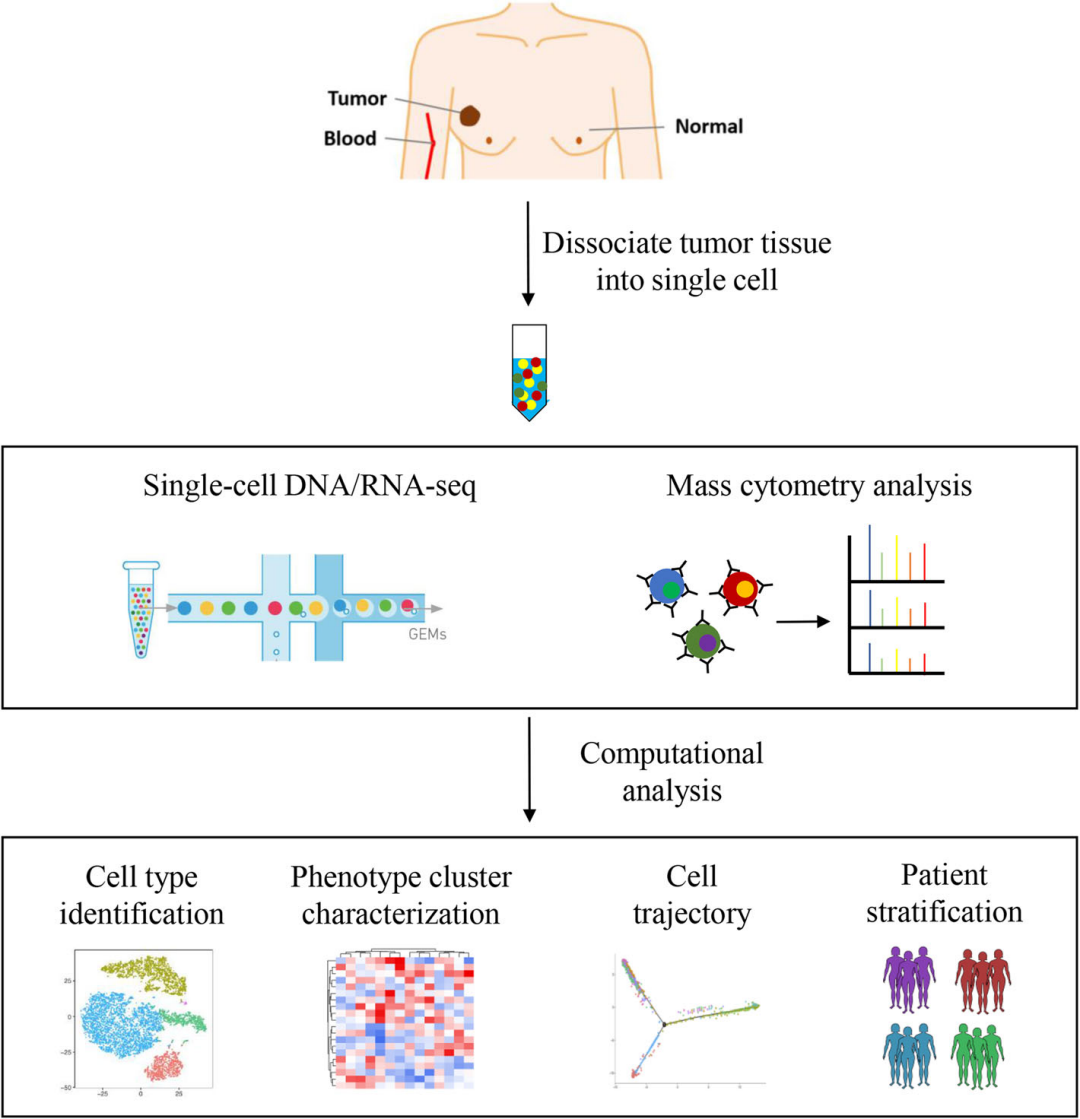
Workflow showing collection and processing of fresh tissues and blood cells for generating single-cell data. Flow chart of experimental design and analysis

There has been a growing interest in characterizing the TME immune landscape at the molecular level [16]. For instance, RNA-sequencing (RNA-seq) of tumor-resident immune cells have helped delineate the cell types and identify the major subsets involved in tumor progression and immune evasion [17]. However, conventional RNA-seq is performed on the RNA extracted from homogenized tissues or bulk cell populations, and only provides an average picture of gene expression levels. Therefore, this approach cannot identify distinct cell types that express specific gene signatures [18, 19]. Single-cell DNA/RNA sequencing (scDNA/RNA-seq) on the other hand allows high-throughput and high-resolution analyses of individual cells. It involves the isolation of single cells, analysis of their genomes or transcripts, and generation of individual sequencing libraries, which can reveal the state and function of single cells (Fig. 2). Recent advances in scDNA/RNA-seq technologies have been able to achieve simultaneous analyses of up to tens of thousands of individual cells [20, 21]. This approach is especially suitable for dissecting the complex breast TME, and assess the heterogeneity of cell populations in and around the breast tumors.

We have reviewed the recent studies on the single-cell expression profiling in breast cancer, which provide novel insights on tumor complexity, improved classification schemes and next generation therapeutic approaches. The summary of current single-cell-based studies for dissecting landscape in breast tumors, is presented in Table 1.

**Table 1:**
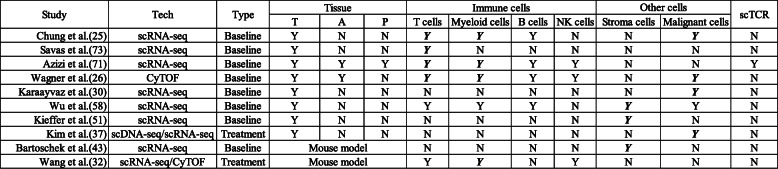
**Summary of current single-cell-based studies for dissecting the landscape in breast tumors**. Bold-italic format: Provide data with in-depth analysis. *Abbreviations*: *tech* technology, *T* tumor, *a* adjacent normal or healthy tissues, *P* peripheral blood, *scTCR* single-cell TCR information, *Y* yes, *N* no.

## Breast cancer cells

Gene expression profiling studies in recent years have identified several cancer-specific markers from bulk tumors for targeted therapy. However, intra-tumoral heterogeneity can influence and even limit the therapeutic response and clinical outcomes to specific targeted therapies [22–24]. ScRNA sequencing allows the assessment of genetic heterogeneity in cancers at single-cell resolution, which can help elucidate the biological complexity of tumors.

Chung et al. analyzed the transcriptomes of 515 single breast tumor cells from 11 patients with the luminal A, luminal B, HER2-positive, and triple-negative (ER/HER2/PR-negative) subtypes [25], and found that cells isolated from HER2-positive tumors were highly diverse and predominantly exhibited the HER2 and triple negative breast cancer (TNBC) phenotypes. In addition, cells isolated from the ER/HER2 double-positive tumors were usually of the ER subtype due to the low expression of HER2 module genes and activation of the ER signaling pathway. Thus, single-cell molecular profiling can help identify ER/HER2 double-positive tumors with prominent activation of the ER pathway, which may benefit from intensive hormone therapy; as well as HER2-positive tumors that may be resistant to targeted therapy due to poor HER pathway activation and higher basal gene expression level. The selection of the appropriate therapeutic regimen can significantly improve patient prognosis. Similarly, Wagner et al. analyzed the proteomes of epithelial cells from tumor tissues [26]. They also found that the tumors of all clinical subtypes consisted of multiple phenotypically distinct cells, although a particular phenotype was usually dominant, reflecting the expansion of the fittest tumor subclone [27]. The phenotypic abnormality scores are higher for the luminal B, luminal B-HER2^+^, TNBC and grade 3 tumor cells compared to the cells of luminal A and lower grade tumors. Phenotype dominance can be particularly important for tumor progression and treatment resistance. For instance, the dominant ERa^−^HER2^−^Survivin^high^ phenotype is associated with resistance to neoadjuvant chemotherapy. Besides, in luminal B tumors, the frequency of ER^+^ cell correlates with that of PD-L1^+^ TAMs and exhausted T cell phenotypes in TME, thus affecting the efficacy of ICB in ER^+^ breast cancer patients. This is in accordance with the previous notion that hormone receptor signaling shapes the breast tumor ecosystem [28].

TNBC is characterized by extensive intra-tumoral diversity, and patients that fail to achieve a pathologic complete response (pCR) to neoadjuvant chemotherapy usually have poor outcomes [29]. This is indicative of the presence of a minor subpopulation of TNBC cells that are resistant to conventional chemotherapy. In agreement with this hypothesis, Karaayvaz et al. identified five distinct epithelial cell clusters within six primary TNBC tumors through scRNA-seq [30], of which cluster 2 had the highest proportion of rapidly cycling cells (40%) that were enriched for cell cycle and DNA repair-related genes. Compared to the other epithelial cells, Cluster 2 cells exhibited the gene signature of luminal progenitors (LPs), which are the possible origin of breast cancers [31], and was functionally characterized by the activation of glycosphingolipid metabolism and associated innate immunity pathways. The notable cluster 2-selective gene included GLTP (glycolipid transfer protein), SPTLC1 (sphingolipid biosynthesis subunit), S1PR1 (sphingosine-1-phosphate receptor), GPI/AMF (glucose-6-phosphate isomerase/autocrine motility factor), the epithelial tight junction assembly factor gene F11R and pro-tumorigenic cytokines CCL20 and CCL22. High expression of the cluster 2 gene signature was related to worse survival outcomes of the TNBC patients, and can therefore be considered as potential prognostic markers and therapeutic targets for TNBC with poor prognosis.

Single-cell analysis also can be used to explore the resistant cell subgroups after anti-tumor treatments, which provided guidance for subsequent anti-tumor therapy. For instance, Wang et al. recently identified a significant IMC subtype that infiltrated in the CDK4/6 inhibitor-resistant tumors. The researchers speculated that combination of IMC-targeting tyrosine kinase inhibitor cabozantinib and ICB may facilitate anti-tumor immunity and reverse the resistance of CDK4/6 inhibitor [32]. Jang et al.analyzed the RNA-seq data of single breast cancer cells and immune cells, and found that the radioresistant tumor cells were associated with a higher rate of PD-L1 positivity and tumor mutation burden (TMB) [33]. In addition, the TNBC cells expressed higher levels of immune checkpoints compared to luminal and HER2-positive subtypes. Their findings suggest that the combination of radiotherapy and ICB can be effective against radiosensitive and PD-L1-overexpressing TNBC cells with higher TMB.

Vu et al. recently developed a novel technique to detect mutations at the single cell level, which can further expand the application of scRNA-seq in cancer research [34]. Specific somatic mutations are associated with clinical efficiency of treatment in breast cancer. For example, GATA3 mutation predicts a better response to aromatase inhibition; PI3K or ERBB2 mutations may sensitize HER2 positive breast tumors to neoadjuvant chemotherapy (NACT) (docetaxel, carboplatin) in association with anti-HER2 treatment (trastuzumab, lapatinib) [35, 36]. Kim et al. combined single-cell DNA and RNA sequencing to profile TNBC samples during NACT to determine whether chemotherapy resistance is caused by the selection of pre-existing clones or via acquisition of new genomic mutations [37]. They found that the resistant genotypes were pre-existing and gradually selected during NACT and transcriptional profiles were reprogrammed in response to chemotherapy.

Taken together, Single-cell sequencing is a promising tool for identifying novel markers associated with TMB or immune checkpoint crosstalk, providing instructions for the clinical medication and predicting individual drug-response with greater accuracy.

## Cancer-associated fibroblasts (CAFs)

Cancer associated fibroblasts (CAFs) are a heterogenous population in the TME, and are classified into distinct subsets on the basis of specific surface markers [38–41]. However, their origin and function in tumor initiation, progression and treatment response are largely unclear. ScRNA-seq have identified different CAF types in breast tumors, which likely originate from distinct parent cells [42, 43], and are mainly involved in the recruitment of immune cells to the tumors and induction of the epithelial–mesenchymal transition (EMT) in tumor cells [42, 44–46]. The CAFs are currently classified into the CAF-S1, CAF-S2, CAF-S3 and CAF-S4 subsets on the basis of specific markers including fibroblast activation protein (FAP), smooth-muscle α actin (SMA), and integrin β1 (CD29). Recent studies on immunocompetent mouse models reported that FAP-expressing CAFs induce an immunosuppressive environment [39, 43, 47–49], and the FAP^hi^ CD29^med-hi^ SMA^hi^ CAFs-S1 in particular promote immunosuppression in the TME by recruiting the CD4^+^CD25^+^FOXP3^+^ Tregs [38, 50].

**Fig. 3:**
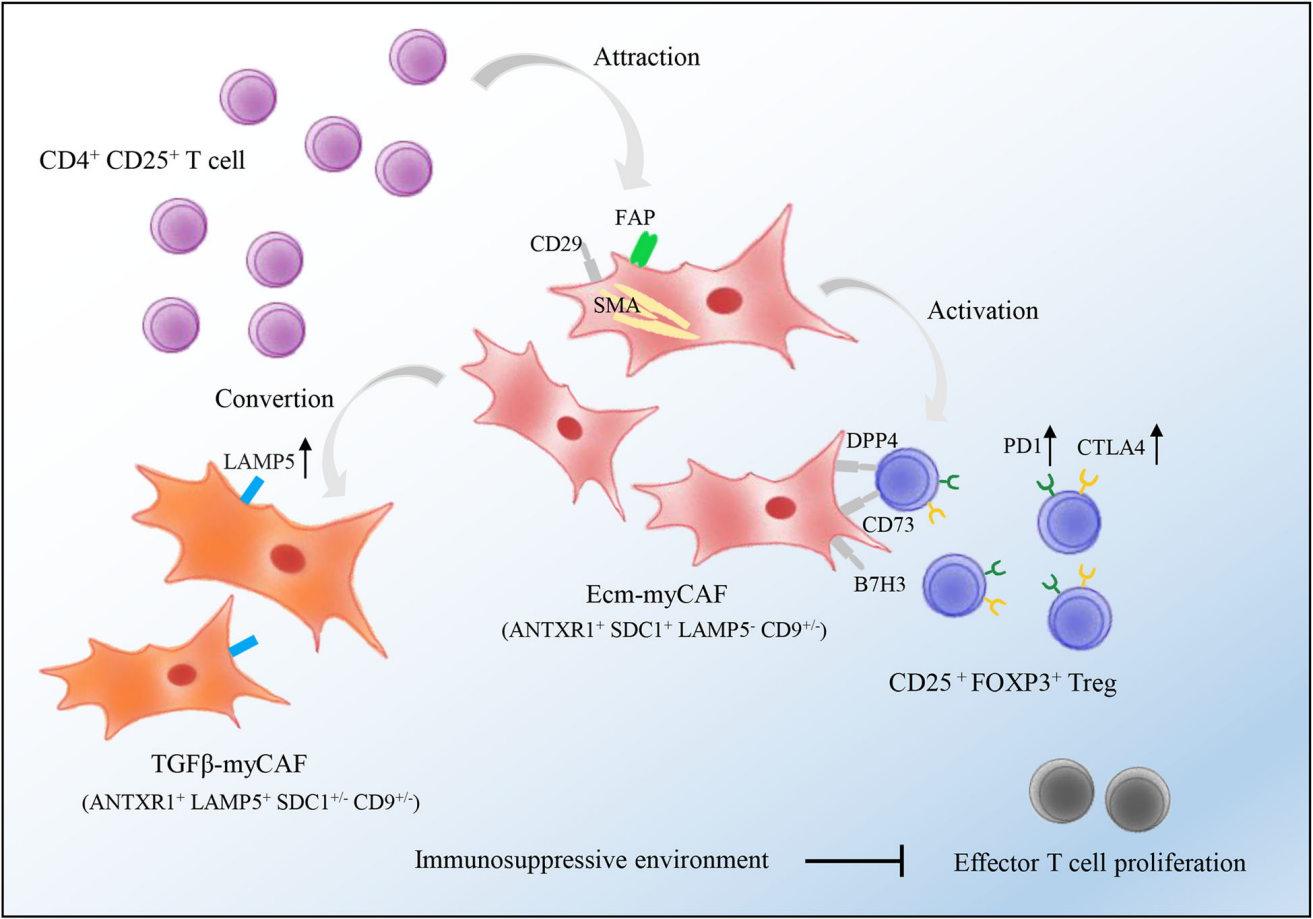
Two CAF subtypes (ecm-myCAF and TGFβ-myCAF) identified in breast cancer by Yann Kieffer [51]**.** A positive feedback loop between the immunosuppressive ecm-myCAF and TGFβ-myCAF CAF-S1 clusters with Tregs likely mediate TME immunosuppression

Kieffer et al. analyzed the gene expression profiles of more than 19,000 FAP^hi^ CAFs-S1 [51] isolated from eight primary breast tumors by scRNA-seq, and identified eight CAF-S1 clusters of which three (1, 2, 5) were inflammatory (“iCAF”) and five (0, 3, 4, 6, 7) were myofibroblastic (“myCAF”). In addition, the different clusters were characterized by high expression levels of genes encoding for extracellular matrix (ECM) proteins (cluster 0, ecm-myCAF), detoxification pathway (cluster 1, detox-iCAF), interleukin (IL) signaling (cluster 2, IL-iCAF), TGFβ signaling pathway (cluster 3, TGFβ-myCAF), wound healing (cluster 4, wound-myCAF), IFNγ (cluster 5, IFNγ-iCAF), IFNαβ (cluster 6, IFNαβ-myCAF), and acto-myosin pathway (cluster 7, acto-myCAF). Both clusters were enriched in TME with a high proportion of PD-1^+^, CTLA4^+^ and TIGIT^+^ CD4^+^ T cells and lower fraction of CD8^+^ T cells. The abundance of two kinds of myCAF subgroups, cluster 0 and cluster 3, was significantly correlated with an immunosuppressive environment. In addition, the cluster 0 increased the expression of PD-1 and CTLA4 at the surface of FOXP3^+^ Tregs, while the CD4^+^ CD25^+^ T cells promoted the conversion of cluster 0 to the cluster 3. This pointed to a positive feedback loop between the immunosuppressive CAF-S1 clusters and Tregs that likely mediate immunotherapy resistance (Fig. 3). In contrast, the cluster 4 correlated with high T cell infiltration, but was enriched in tumors that did not respond to immunotherapy. Therefore, the cluster 4 is a potential surrogate marker of primary resistance to immunotherapies in tumors with high T cell infiltration. Taken together, CAF-S1 profiling during breast cancer diagnosis can augment the predictive value of infiltrating CD8^+^ T cells and Tregs amounts in distinguishing between responder and non-responders. Combination of PD-1 and/or CTLA4 blockade with CAF-S1 targeting should be further explored in breast cancer.

Besides, there are many other kinds of classifications of CAFs. Bartoschek et al. delineated the heterogeneity of 768 CAFs isolated from the genetically engineered MMTV-PyMT mouse model of breast cancer, and defined three transcriptionally distinct subpopulations: vascular CAFs (vCAFs), matrix CAFs (mCAFs) and developmental CAFs (dCAFs) [43]. In addition to differential gene expression profiles that correspond to different functions, the CAF subsets showed unique spatial location within the tumor parenchyme. ①The vCAFs express high levels of vascular regulators such as Notch3, Epas1, Col18a1 and Nr2f2, and share several pericyte markers including Cspg4, Rgs5, Pdgfrb, Des and Cd248 [52]. This is consistent with a previous report that showed a significant correlation between the vCAF, endothelial and microvasculature gene signatures [53, 54]. A nested case–control study comparing the transcriptional data of metastatic and non-metastatic breast tumors found that the vCAF gene signature was an independent risk factor of distant metastasis [55]. Furthermore, co-immunostaining for the vCAF marker Nidogen-2 and the endothelial cell marker CD31 showed that the Nidogen-2+ vCAFs were predominantly localized around the blood vessels in the early stages of tumor development, which is consistent with their gene expression profile. In the more advanced tumors, however, the Nidogen-2-positive cells had mostly detached from the vasculature and infiltrated the tumor stroma. The enrichment of vCAFs in the tumor core rather than the peri-tumoral region strongly indicates that hypoxia fuels the detachment of vCAFs from their perivascular niche. Based on the gene expression and spatio-localization data, we speculated that the vCAFs originate from a pool of perivascular cells and gradually invade the tumor stroma over the course of tumor growth. ②The mCAFs exhibit gene signatures associated with stroma-derived invasion and stroma-related treatment outcome [56, 57], including the ECM-related genes such as glycoproteins (Dcn, Lum, and Vcan), structural proteins (Col14a1), matricellular proteins (Fbln1, Fbln2, and Smoc), and matrix-modifying enzymes (Lox and Loxl1). In addition, mCAFs also express high levels of the immune cell-attracting factor CXCL14, suggesting a regulatory role in the anti-tumor immune response. Furthermore, the Fibulin-1 and PDGFRα expressing mCAFs are abundant in the invasive front of tumors as opposed to the tumor core. The PDGFRα-positive mCAFs in particular reside predominantly within collagen-rich stroma, in keeping with their putative role as ECM producers. In contrast to the vCAFs, the relative number of mCAFs is known to decrease during tumor progression. Thus, based on their marker expression and peripheral location in the tumors close to the surrounding normal tissue, mCAFs likely originate from resident fibroblasts that are eventually incorporated into the tumor. ③In addition to the ECM genes like Slug, Snail and Twist1, dCAFs also express the stem cell-related gene signature (Scrg1, Sox9 and Sox10), indicating a role in tissue development. Immunostaining of human tumor tissues confirmed that SCRG1-positive dCAFs co-localized with the malignant epithelium during early stages of tumor development, and were present in the epithelium as well as fibrous stroma of more advanced tumors. This suggests that dCAFs originate as malignant epithelial cells that secrete paracrine signaling molecules. Intriguingly, the oncogene PyMT is highly expressed in dCAFs, which further underscores their malignant cell origin. Furthermore, dCAF genes have been detected in both tumor epithelial and stromal mesenchymal cells, indicating that they may originate from tumor cells that have undergone EMT. The dCAF gene signature is therefore a potential biomarker for distinguishing the EMT cells from the non-EMT malignant cells and mesenchymal stromal cells.

Wu et al. analyzed the transcriptomes of single cells isolated from five TNBC samples, and found that stromal heterogeneity diverged into four distinct states: myCAFs, iCAFs, and the differentiated perivascular-like (dPVL) and immature PVL (iPVL) cells [58]. Contradicting with the findings of Bartoschek et al. above, they detected dCAF marker genes such as Scrg1, Sox9 and Sox10 exclusively in cancer epithelial clusters that are classified based on keratin expression. This suggests that murine “dCAFs” of EMT origin are either unique to mouse models or are simply cancer cells with low EPCAM level, which is used for negative CAF isolation [59, 60]. The iCAFs clusters on the other hand expressed an array of immunomodulatory molecules that can bind to cognate receptors on T cells. This is consistent with a previous study that implicated CAFs in the recruitment and activity of Tregs through CXCL12, CD40, B7H3, DPP4 and CD73 [38]. In the context of clinical relevance, iCAF gene signatures were strongly associated with CTL dysfunction in the TNBC patient cohorts. Some other studies have also shown that iCAFs express the complement C5, IL6 and TGFβ, which are known to regulate myeloid cells [61, 62]. PVL cells, described by Bartoschek as “vascular-like CAFs” on account of the expression of vascular development markers such as CD146, are phenotypically distinct from the fibroblast lineage since they lack the defining fibroblast trait of collagen deposition. They are characterized by perivascular markers including MCAM (CD146), CAV1, RGS5, MYH11 and TAGLN (SM-22-Alpha), and several CAF-related markers like ACTA2 (⍺-SMA), PDGFRB, THY1 (CD90), S100A4 (FSP-1) and ITGB1 (CD29) [38, 63, 64]. Based on their surface phenotype, it is possible that PVL cells originate from vascular smooth muscle cells (VSMCs). One study showed that PVL cells rather than CAFs are predictive of the ER-targeted therapeutic response in ER-positive breast cancers [65]. In addition, the dPVL stromal subset strongly correlated with CTL exclusion, and patients with higher dPVL numbers had consistently lower tumor infiltrating lymphocytes (TILs) and CD8+ T cell counts compared to patients with fewer PVL cells and similar tumor pathological classification. Considering that PVL cells likely detach from the vascular structure, the correlation above may be indicative of reduced lymphocyte extravasation from dysregulated tumor blood vessels [66, 67] and lower vascular integrity. Thus, the dPVL profile is a potential biomarker for identifying patients suitable for vessel-targeted therapeutic strategies. Taken together, the iCAF and dPVL subsets are strongly associated with immune evasion in multiple independent TNBC cohorts, and are promising therapeutic targets.

Taken together, the cellular resolution of CAFs were significantly improved by scRNA-seq. It provided compelling evidence for the existence of at least three spatially and functionally distinct subsets of breast CAFs. The iCAF, vCAF and mCAF signatures were highly conserved in breast tumors samples and held prognostic capabilities by their association to CTL dysfunction or metastatic dissemination. Further, the mCAF signature was correlated to a treatment-predictive stromal signature, opening up the possibility for development of novel targeted drugs or biomarkers of clinical significance with increased precision.

## Immune cells

Studies increasingly show that immune cells play an important role in tumor progression and therapeutic response [68]. The considerable variation in the efficacy of immunotherapies targeting CTLA-4 and PD-1 has been attributed to the heterogeneity of immune cells across tumors. The T cells and myeloid cells are the most abundant subsets in tumors, and are therefore clinically most relevant. The Treg cells that overexpress the transcription factor Foxp3 and inhibit effector T cells are markedly increased in solid tumors [69]. Likewise, the proportion of the regenerative M2 macrophages that promote tumor growth and metastasis is relatively higher among the TAMs compared to the pro-inflammatory M1 macrophages that inhibit tumor progression [70]. In addition, B cells, NK cells, DC cells etc. also play important roles in the TME. However, whether the immune cell states differ between the normal and tumor tissues, or whether they represent a limited number of discrete differentiation or activation intermediates remain to be elucidated. Alternatively, these cell states may occupy a single contiguous spectrum shaped by the TME. Extensive scRNA-seq analyses can further dissect these immune cell subsets in tumors and para-tumor tissues.

### T cells

The number and types of T cells infiltrating breast tumors are a key factor determining the therapeutic and prognostic outcomes, particularly for the TNBC patients. The intra-tumoral T cell population is heterogeneous with a frequent enrichment of Tregs, along with the transformation of CD8^+^ T cells to the exhausted or tissue resident memory (T_RM_) phenotype. The anti-inflammatory, exhaustion, hypoxia and anergy genes are up-regulated, and the immunotherapy target genes are differentially expressed in these TILs. Therefore, the T cell landscape in tumors is a promising biomarker for prognostic subtyping and treatment monitoring.

Azizi et al. analyzed the immune environment of eight primary breast carcinomas (BC), including one HER2-positive, two TNBCs and five ER-positive tumors, at the single cell level and detected considerable heterogeneity within each tumor. In addition, the cellular composition was associated with the resident tissue [71]. For instance, the CTLs, Treg cells and activated TAMs were more abundant in the tumors relative to the normal tissues, whereas the naive T cells were strongly enriched in blood-specific clusters and the B cells were more prevalent in the lymph nodes. Furthermore, the intra-tumoral T cells were constitutively activated, and existed as multiple transient populations across the differentiation spectrum rather than a few discrete and stable states. T cell activation and differentiation was accompanied by the upregulation in genes encoding for cytolytic granzymes A and K (GZMA and GZMK), proinflammatory cytokines (IL-32), cytokine receptor subunits (IL2RB), and chemokines (CCL4, CCL5) and their receptors (CXCR4, CCR5). In addition, the co-stimulatory (CD2, GITR, OX40 and 4–1BB) and co-inhibitory receptors (CTLA-4 and TIGIT) showed the strongest correlation with T cell terminal differentiation. Tumor-resident T cells are exposed to varying degrees of inflammation, hypoxia and nutrient deprivation. While the responses to many of these stimuli individually correspond to phenotypic continuums, their combinations may result in more discrete cellular states. For instance, the CD4+ effector and central memory clusters expressed varying levels of genes involved in type I and II interferon response, hypoxia and anergy, whereas the CD8+ effector and central memory clusters showed differential expression of activation, pro-inflammatory and cytolytic effector pathway-related genes. Taken together, the T-cell clusters in BC are characterized by diverse environmental signatures, tumor-resident T cells are mapped on the continuous activation and differentiation trajectories, and combinatorial environmental stimuli and T cell receptor (TCR) utilization shape more diverse phenotypes of TILs. These findings provide some novel insights into the phenotypic diversity for breast tumor-infiltrating immune cells, which might facilitate a better understanding of the mechanisms of cancer progression and therapeutic responses.

Chung et al. recently found that the immune cell subpopulations infiltrating primary breast tumors comprised mainly of T cells, B cells and TAMs [25]. Tumor-resident T cells exhibit distinct naive, co-stimulatory, regulatory, exhaustion and cytotoxicity gene signatures [25, 72]. The luminal B-type tumors harbor T cells with naive/early co-stimulatory signatures at the primary sites, whereas the co-stimulatory signature is prevalent in the lymph nodes. In contrast, the HER2-positive and TNBC tumors are populated with T cells expressing regulatory markers such as IL2RA (also known as CD25). In addition, T cell populations with a predominant exhaustion signature and both exhaustion and cytotoxicity signatures have also been detected in TNBC. T cells expressing high cytokine and chemokine levels have also been detected in the immune cell infiltrates from TNBC tumors, which exhibit the immuno-suppressive exhausted or regulatory phenotypes [45, 72]. T cells with a strong exhaustion signature are suitable targets of ICB. Although these cells express only modest levels of PD-1, other inhibitory receptors such as TIGIT and LAG3 have been frequently detected and are potential targets for checkpoint inhibition.

Savas et al. profiled 6311 T cells isolated from human breast cancer tissues, and identified a new subclass of tissue-resident memory CD8^+^ T cells (T_RM_) [73]. The T_RM_ gene signature was more abundant in the TNBC tissues than in other BC subtypes. In the study of Azizi et al., the majority of CD3^+^CD8^+^ TILs in BC were of the T effector memory (T_EM_, CCR7^−^CD45RA^−^) and effector memory re-expressing CD45RA (T_EMRA_, CCR7^−^CD45RA^+^) phenotypes [71]. This new CD3^+^CD8^+^ T cell subset expressed integrin subunit alpha E chain (ITGAE) or CD103, with low expression of “tissue egress genes” such as KLF2, SELL, S1PR1, S1PR5 and KLRG1 [74, 75]. The TCR repertoires of the T_RM_ and T_EM_ cells do not overlap, indicating distinct antigen specificities. In fact, the TCR repertoire has been shown to be at least partly responsible for the heterogeneity of TILs subpopulations found in BC. The T_RM_ population was also characterized by high expression of genes encoding for effector and cytotoxic proteins such as GZMB and perforin (PRF1), and the immune checkpoint molecules such as HAVCR2 (TIM3), PDCD1 (PD1), CTLA4, TIGIT and LAG3. In addition, abundance of CD3^+^CD8^+^ T_RM_ cells has been associated with improved prognosis and longer overall survival (OS) in early-stage TNBCs [73, 76]. Thus, Savas et al. hypothesized that the CD3^+^CD8^+^CD103^+^ T_RM_ cells are a potentially relevant target for ICIs, and a higher frequency of the T_RM_ cells in advanced BC patients is likely predictive of a better response to anti-PD-1 therapy [77].

Wagner et al. deciphered the immune landscape in different BC subtypes using single-cell proteomics [26], and found that consistent with transcriptional analysis, the tumor-associated T cell clusters existed as a phenotypic continuum across CD4^+^ and CD8^+^ T cells and most of them had an CD197^low^CD45RA^low^ effector memory phenotype [71, 78]. The PD-1^+^ T cells comprised 26.6% of all the tumor-infiltrated T cells, and were mainly clustered within the CD8^+^ lineages. However, the mean expression level of PD-1 in the CD8^+^ T cells was lower than CD4^+^ T cells. The PD-1^high^CD8^+^ T cells also expressed other co-inhibitory markers (TIM-3, CTLA-4) and activation receptors (HLA-DR, CD38). In contrast, the PD-1^high^CD4^+^ T cells expressed CTLA-4 and CD38 while TIM-3 and HLA-DR were negative. Both PD-1^int^CD8^+^ and PD-1^int^CD4^+^ T cells were negative expression of TIM-3, CTLA-4, HLA-DR, and CD38. The mean expression level of PD-1 was correlated to the frequency of PD-1^+^ T cells in the CD4^+^ and CD8^+^ compartments, indicating that these cells result from T cell expansion. In addition, a higher frequency of Tregs and exhausted T cells were observed in ER^−^ and high-grade ER^+^ tumors, possibly indicating the cellular basis of better response to ICIs in these patients. A better understanding of the tumor–immune relationships in the BC ecosystem can improve patient stratification for immunotherapy.

### Myeloid cells

The myeloid lineage is a heterogeneous population including granulocytes and mononuclear phagocytes that play critical roles in anti-tumor immunity [79, 80]. Mononuclear phagocytes like monocytes, macrophages and dendritic cells (DCs) also function in innate immunity by recognizing and engulfing pathogens, and perform an ancillary role in adaptive immunity by presenting antigens to T cells [79]. However, little is known regarding the myeloid compartment in breast TME. Neutrophils are the most common subtype of granulocytes, and typically participate in the innate responses against bacterial and fungal infections. However, their roles in tumor immunity remain controversial [81]. Ponzetta et al. found that neutrophils were essential for the polarization of a subset of unconventional T cells with an innate-like phenotype, and thus promoted the anti-tumor immune response [82]. In addition, Fridlender et al. identified distinct N1 and N2 neutrophil phenotypes in the TME that respectively corresponded to anti-tumorigenic and pro-tumorigenic functions [83]. These findings underscore the functional diversity of tumor-associated neutrophils (TANs), and warrant further research which might open new opportunities for regulating neutrophils as a mode of cancer therapy.

Macrophages are a heterogeneous population of phagocytic cells with complex phenotypic and functional properties. The TAMs can polarize to the classically activated (M1) or alternatively activated (M2) phenotypes depending on specific stimuli to respectively exert immunostimulatory or immunosuppressive functions [84]. The pro-inflammatory M1 macrophages have tumor-killing capacity, whereas M2 macrophages promote immune suppression and tumor progression [7] by secreting anti-inflammatory cytokines [25, 85]. However, single cell profiling studies increasingly show that TAM behavior is not in accordance with the conventional polarization model, in which a positive correlation between M1 and M2 gene signatures was showed [26, 71, 86, 87]. Even so, the influence of TAMs on the TME remains unclear, warranting further investigation to be a new class of immunotherapy targets. Myeloid-derived suppressor cells (MDSCs) are a heterogeneous population of myeloid precursors that resemble immature neutrophils [88, 89]. MDSCs are recruited to the tumor site by the cancer cell-secreted growth factors and pro-inflammatory cytokines like granulocyte-macrophage colony stimulating factor (GM-CSF), VEGF and CCL3/4/5 [90–94]. After expanding in the TME, MDSCs induce NK cell and T-cell anergy, angiogenesis and EMT [95–97], thereby suppressing both innate and adaptive anti-tumor immunity [98, 99]. Tumor-activated MDSCs also infiltrate into the peripheral normal organs and establish a premetastatic niche by supporting metastatic cell seeding and survival, and suppressing immune rejection [100]. Clinically, increased circulating MDSCs correlate with poor patient prognosis and survival [101].

DCs are the key APCs and can be classified into the plasmacytoid DCs (pDCs) and conventional DCs (cDCs). The pDCs produce high levels of type I interferon and thus play an important role in modulating innate and adaptive immunity [102]. Although pDCs were originally recognized for their roles in antiviral immunity, recent studies show a complex role in tumor immunity as well [103–107]. The cDCs are classified into the cDC1s and cDC2s subtypes that differ in terms of phenotype, function and transcriptional factor dependency [108]. While cDC1s present antigens on MHC class I molecules to CD8^+^ T cells, the cDC2s function as APCs to CD4^+^ T cells via MHC class II receptor [109]. Furthermore, cDC1s promote local immune responses within the TME by delivering antigens to the tumor-draining lymph nodes (dLNs) [110, 111]. The cDC2s on the other hand are highly abundant and heterogeneous, and promote a wide range of CD4^+^ T cell mediated immune responses [112–114]. Taken together, the pDCs and cDC1s are relatively homogeneous populations with well-established functions, although their roles in anti-tumor immunity need further verification. In contrast, the functional roles of the heterogenous cDC2s remain elusive.

Based on the research above described, the researchers further explored myeloid compartment in breast TME through single-cell analysis. Wagner et al. analyzed 144 human breast tumor and 50 non-tumor tissue samples by using single-cell proteomics, identifying the following five myeloid clusters: (1) CD14-expressing classic (M_06_, CD14^+^CD16^−^) and inflammatory monocytes (M_15_, CD14^int^CD16^+^), (2) early immigrant macrophages (M_03_, M_11_, M_13_, HLA-DR^int^CD192^+^), (3) tissue-resident macrophages (M_08_, M_09_, M_16_, CD206^+^HLA-DR^int^), (4) TAMs (M_01_, M_02_, M_04_, M_14_, M_17_, CD64^high^HLA-DR^high^), and (5) MDSCs (M_07_, M_10_, M_12_, HLA-DR^−/low^) [26]. Tumors were rich in TAMs and short of M_06_, M_08_, M_09_ and M_15_ phenotype compared with para-carcinoma tissue. Myeloid cells in tumor were phenotypically heterogeneous and more than 10% of them were PD-L1 positive. For example, the M_01_ TAMs expressed CD38, which is associated with immunosuppressive macrophages and MDSC-mediated T cell suppression. M_01_ TAMs also expressed other pro-tumor markers (CD204, CD206, CD163) and the anti-tumor marker CD169. In addition, the M_02_ TAMs expressed CD38, CD204, CD163 and CD169whilethe M_17_ TAMs expressed CD38 and CD169 [86, 115]. These findings linked CD38 and PD-L1, confirming the co-expression of pro- and anti-inflammatory markers on the TAMs [71, 86]. It also showed that PD-L1^+^ TAMs were enriched in grade 3 breast tumors compared to grade 2 tumors, suggesting that TAM infiltration is associated with tumor aggression [6]. Furthermore, ER^−^ tumors and luminal B tumors are more likely comprised of M01,M07 or M17lineage, with a higher frequency of PD-L1^+^ TAMs compared to luminal A tumors. Thus, hormone receptor signaling may be a major factor in shaping the tumor ecosystem [28].

Chung et al. dissected the TAMs according to CD14 and CD68 expression, as well as phagocytic enzymes associated with macrophage function [25, 116]. The putative TAM populations expressed several M2-type genes such as CD163, MS4A6A and TGFBI, in addition to pro-tumorigenic and pro-angiogenic genes like PLAUR13 and IL-8 [25]. It is consistent with the previously reported immunosuppressive M2 rather than activating M1 signature of TAMs [117]. Another scRNA-Seq study identified four major branches of intra-tumoral myeloid cells [71]. The first branch consisted predominantly of TAMs expressing APOE, CD68, TREM2 and CHIT1, which correlate with differentiation and activation of macrophages. In addition, the M2 phenotype genes such as MARCO, NRP2 and CD276 were upregulated in these cells, as were the immunostimulatory genes of M1 macrophages, including chemokine CCL3. The same cells usually expressed both M1 and M2 associated genes which were positively correlated with one another along the cell growth and development trajectory [118]. These findings challenge the established M1/M2 polarization model wherein M1 and M2 activation states exist as mutually exclusive discrete states. Instead, this study demonstrates the co-existence of M1 and M2 states that are positively correlated with each other [119], which reiterates the findings of bulk TAM analysis in mouse models of oncogene-driven breast cancer, and mass cytometry analyses of myeloid cells in other cancer tissues [86, 120, 121].

### Natural killer (NK) cells

NK cells are prototypical innate lymphoid cells that exert cytotoxic functions without MHC specificity, and thus complement the MHC-restricted tumor cells by CTLs [122]. They can eradicate tumor cells directly by producing cytolytic granules, or indirectly by activating other immune cells via secretion of proinflammatory cytokines and chemokines [123, 124]. NK cell activation is mediated by the combined action of stimulatory and inhibitory receptors. The inhibitory receptors interact with MHC class I molecules expressed on normal cells and contribute to the self-tolerance, whereas the stimulatory receptors sense the loss of MHC class I, leading to NK cells activation [123]. However, emerging studies show that NK cells in the TME have reduced cytotoxic activity and lower proinflammatory cytokines expression. Böttcher et al. discovered that tumor-resident NK cells recruited cDC1 cells to facilitate anti-tumor immunity, while tumor cells released prostaglandin E2 which impaired NK cell function, leading to immune evasion [124]. Therefore, NK cells are also potential targets for immunotherapy. However, little is known regarding their role in the breast TME at the single cell level. One study recently identified CD56^−^ and CD56^+^ NK cell clusters in the TME without further analysis [71]. Therefore, more studies are needed to explore the phenotype, function and interactions of NK cells in the TME at the single-cell level.

### B cells

B cells are the mediators of adaptive humoral immunity, and differentiate into memory B cells or immunoglobulin (Ig)-secreting plasma cells in response to antigenic stimulation. The antibodies produced by the activated B cells bind to and neutralize the target antigens [125]. In addition, the B cells also contribute to T cell responses by functioning as APCs or regulatory cells, and secreting immunomodulatory cytokines [126]. Furthermore, B cells also maintain secondary lymphoid organ architecture and facilitate the formation of tertiary lymphoid structures (TLSs) consisting of immune cell aggregates at the tumor sites for long-term immunity [127, 128].

Tumor-infiltrating B cells (TIBs) exhibit considerable phenotypic and functional diversity [129]. Chung et al. classified 175 immune cells in breast TME into three groups, of which the largest group consisted of B cells expressing immunoglobulins and B-cell-specific transcriptional factors. Most of these cells originated from the tumor-infiltrating lymph nodes, and were further classified into two subclasses – one with an expression signature of centroblasts/centrocytes and the other with that of naive B lymphocytes [130]. Wagner et al. analyzed the B cells in human breast tumor tissues using mass cytometry and found that B cell clusters were more prevalent in the lymph node than in other tissues.

TIBs can promote tumor progression by inhibiting T-cell mediated immune responses through paracrine mediators that act on myeloid cells or cancer cells [131]. However, the anti-tumor immune responses of B cells induced by chemotherapy in breast cancer were recently analyzed using the scRNA-seq. Lu et al. performed scRNA-seq of tumor-infiltrating B cells from paired pre- and post-NACT breast tumor samples [132], and found that a distinct B cell subtype with high-expressed inducible T-cell co-stimulator ligand (ICOSL) was significantly expanded after NACT. Furthermore, the amount of ICOSL+ B cell was significantly increased in patients with partial or complete remission compared to stable or progressive diseases, indicating that this subset is also correlated with improved therapeutic effect. Beyond that, an enrichment of ICOSL+B cell was an independent positive prognostic factor for disease-free survival and OS of breast cancer. The mechanism underlying chemotherapy-mediated B cell switch was further investigated in a mouse model, which revealed that the production of ICOSL+B cells post-chemotherapy, and the anti-tumor immune response elicited by these cells was dependent on complement receptor type 2 (CR2) signaling. In addition, CD55 expressed in tumor inhibited the induction of complement-dependent ICOSL+ B cell and dampened anti-tumor immunity, thus become another potential target to enhance anti-tumor immune response. Hollern et al. found that ICIs induced the activation of T follicular helper cells and B cells in TNBC, and the activated B cells facilitated the anti-tumor response by secreting antibodies and presenting antigens to the T cells [133]. Thus, B cell-driven activation of T cells, as the mechanism underlying the response to checkpoint inhibitors, could be induced to enhance the efficacy of ICB.

These findings underscore the crucial roles of B cells in anti-tumor immune regulation, although further investigation is needed to uncover the underlying mechanisms [127, 134].

In conclusion, single-cell analysis revealed continuity of differentiation states and expansions of a “phenotypic space” as principal features of the two main cellular targets of cancer immunotherapy - T cells and myeloid cells. These observations will facilitate better understanding of potential mechanisms behind immune cell contributions to promoting and opposing tumor progression. Besides, the roles of MDSCs, DCs and NK cells in anti-tumor immunity remain elusive. Identification of the gene expression features and functions of them by scRNA-Seq was needed in future. The summary of crucial cellular components in breast cancer TME were described in Table 2.

**Table 2:** Summary of crucial cellular components in breast cancer TME described in this review

Cell Types	Classification		Molecular markers	Functions or prognostic values	Study
Breast cancer cells	All		ERα^−^HER2^−^Survivin^high^	Significantly correlated with resistance to neoadjuvant chemotherapy	Wagner et al. [26]
	TNBC	“cluster 2”	GLTP, SPTLC1, S1PR1, GPI/AMF, F11R, CCL20, CCL22	Significantly correlated with worse survival outcomes of the TNBC	Karaayvaz et al. [30]
CAFs	myCAF	“cluster 0, 3, 4, 6, 7”	Extracellular matrix (ECM) proteins; TGFβ signaling pathway; wound healing; IFNαβ; acto-myosin pathway	Significantly correlated with an immunosuppressive environment	Kieffer et al. [51]
	mCAF		Glycoproteins (Dcn, Lum, and Vcan); structural proteins (Col14a1); matricellular proteins (Fbln1, Fbln2, and Smoc); Matrix-modifying enzymes (Lox and Loxl1); CXCL14; Fibulin-1; PDGFRα	The relative number of mCAFs decreased during tumor progression	Bartoschek et al. [43]
	vCAF		Notch3, Epas1, Col18a1, Nr2f2, Cspg4, Rgs5, Pdgfrb, Des, Cd248	An independent risk factor of distant metastasis	Bartoschek et al. [43]
	dPVL		Perivascular markers including MCAM (CD146), CAV1, RGS5, MYH11, TAGLN (SM-22-Alpha); ACTA2 (⍺-SMA), PDGFRB, THY1 (CD90), S100A4 (FSP-1), ITGB1 (CD29)	Strongly correlated with CTL exclusion; A potential biomarker for identifying patients suitable for vessel-targeted therapeutic strategies	Wu et al. [58]
	iCAF		CXCL12, CD40, B7H3, DPP4, CD73, C5, IL6,TGFβ	Strongly associated with CTL dysfunction and myeloid cells regulation	Wu et al. [58]
Immune cells	CD8 + T cells	T_RM_	ITGAE, CD103, GZMB, perforin (PRF1), HAVCR2 (TIM3), PDCD1 (PD1), CTLA4, TIGIT and LAG3; low expression of KLF2, SELL, S1PR1, S1PR5 and KLRG1;	Associated with improved prognosis and longer OS in early-stage TNBC; A predictor of a better response to anti-PD-1 therapy	Savas.et al. [73]
	Myeloid cells	TAM	PD-L1+ CD64^high^HLA-DR^high^	Associated with tumor aggression	Wagner et al. [26]
	B cell		ICOSL+	Correlated with improved neoadjuvant chemotherapy effect; An independent positive prognostic factor for DFS and OS of breast cancer.	Lu et al. [132]

*Abbreviations*: *TNBC* triple negative breast cancer, *dPVL* differentiated perivascular-like, *DFS* disease-free survival, *CAF* Cancer-associated fibroblast, *TRM* Tissue-resident memory, *TAM* Tumor-associated macrophages, *CTL* Cytotoxic T lymphocytes

## Conclusion

There is an urgent need to identify novel prognostic biomarkers and therapeutic targets for breast cancer in order to improve patient prognosis and survival. Single-cell detection techniques including scDNA-seq, scRNA-seq and mass cytometry have helped dissect the complex breast cancer microenvironment and revealed distinct cell subpopulations that influence the anti-tumor response, and are therefore potential therapeutic targets.

By using Single-cell detection, researchers have found that the endocrine resistance of fulvestrant and tamoxifen was caused by a group of pre-existing genetically distinct cells not sensitive to endocrine therapy and these cells were highly selected during treatment [135]. Therefore, the innovation of novel drugs targeting these cells might reverse the endocrine resistance. Single-cell detection could also be used to detect the emergence of resistant cell subsets after treatment, which could have significant clinical implications for second-line treatment decision-making on available or new target drugs. Collectively, integrating Single-cell detection into basic and translational research could promote personalized therapy by identifying potential treatment targets to develop novel drugs and reveal promising biomarkers to monitor treatment efficacy and guide therapeutic decision-making.

We have reviewed the recent progress in analyzing the landscape of breast cancer TME, which will be a valuable resource for identifying clinically relevant cells for patient stratification and precision therapy.

## Data Availability

Not applicable.

## Abbreviations

TME: Tumor microenvironment
scDNA-seq: Single-cell DNA sequencing
scRNA-seq: Single-cell RNA sequencing
ER: Estrogen receptor
HER2: Human epidermal growth factor receptor 2
PI3K: Phosphatidylinositol 3-kinase
AKTs: AKT serine/threonine kinases
mTOR: Mammalian target of rapamycin
PARP: Poly (ADP-ribose) polymerase
CDK4: Cyclin dependent kinase 4
TAMs: Tumor-associated macrophages
Tregs: Regulatory T cells
CTLs: Cytotoxic T lymphocytes
PD-L1: PD-1 ligand
ICIs: Immune checkpoint inhibitors
ICB: Immune checkpoint blockade
PR: Progesterone receptor
CAFs: Cancer-associated fibroblasts
EMT: Epithelial–mesenchymal transition
FAP: Fibroblast activation protein
SMA: Smooth-muscle α actin
ECM: Extracellular matrix
vCAFs: Vascular CAFs
mCAFs: Matrix CAFs
dCAFs: Developmental CAFs
TNBC: Triple negative breast cancer
dPVL: Differentiated perivascular-like
iPVL: Immature PVL
VSMCs: Vascular smooth muscle cells
TILs: Tumor infiltrating lymphocytes
pCR: Pathologic complete response
LPs: Luminal progenitors
TRM: Tissue resident memory
BC: Breast carcinomas
TCR: T cell receptor
OS: Overall survival
DCs: Dendritic cells
TANs: Tumor-associated neutrophils
MDSCs: Myeloid-derived suppressor cells
IMC: Immature myeloid cells
GM-CSF: Granulocyte-macrophage colony stimulating factor
dLNs: Tumor-draining lymph nodes
NK: Natural killer
TLSs: Tertiary lymphoid structures
TIBs: Tumor-infiltrating B cells
TMB: Tumor mutation burden
NACT: Neoadjuvant chemotherapy
ICOSL: Inducible co-stimulator ligand
